# Dual‐pronged genome engineering: Enhancing crop resistance through elite allele pyramiding and susceptibility gene editing

**DOI:** 10.1002/imo2.70030

**Published:** 2025-06-04

**Authors:** Qingdong Zeng, Zhensheng Kang

**Affiliations:** ^1^ State Key Laboratory for Crop Stress Resistance and High‐Efficiency Production, College of Plant Protection Northwest A&F University Yangling Shaanxi China

## Abstract

Developing durable crop disease resistance remains a primary breeding objective, achievable through pyramiding resistance genes and editing susceptibility (S) genes. A recent study identified 10 stable Verticillium wilt resistance genes and eight negative regulators via integrated genome‐wide association studies and transcriptome‐wide association studies‐eQTL analyses in upland cotton. Both pyramiding resistance alleles and CRISPR‐mediated knockout of S‐genes significantly reduced disease severity. These findings enable rapid implementation of disease resistance improvement in the genomics era.

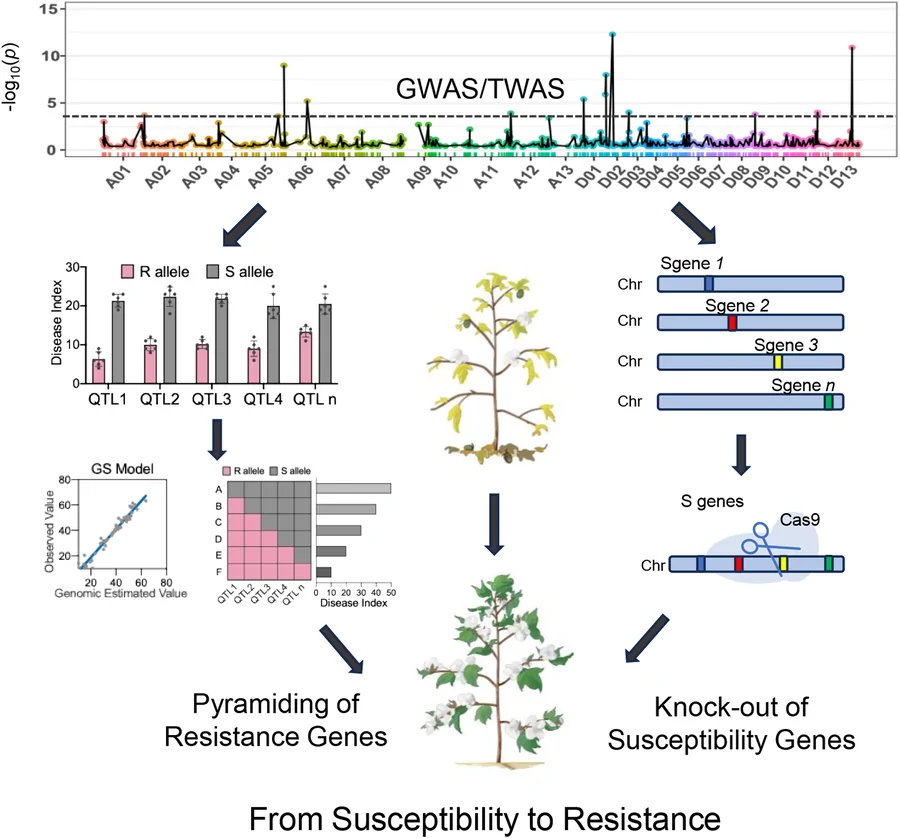

Global yield losses (17%–30%) in key crops (wheat, rice, maize, potato, and soybean) driven by 137 pathogens/pests disproportionately impact food‐insecure hotspots with rapid population growth [1]. As crop diseases cause substantial yield losses globally, resistant cultivars remain the optimal disease control strategy. However, resistance conferred by a single resistance gene tends to be easily overcome by genetic changes in pathogens/pests. To address this problem, two strategies were proposed. Firstly, the concept of gene pyramiding was introduced, which is a systematic breeding strategy that integrates multiple desirable genes from diverse parents into a single genotype [2]. Consequently, modern breeding strategies emphasize the benefits of pyramiding multiple resistance genes to develop varieties with more durable resistance, particularly including less effective quantitative resistance (partial resistance) genes (Figure 1). However, beyond enhancing resistance through pyramiding genes, an alternative approach involves targeting susceptibility (S) genes to disrupt pathogen compatibility.

**FIGURE 1:**
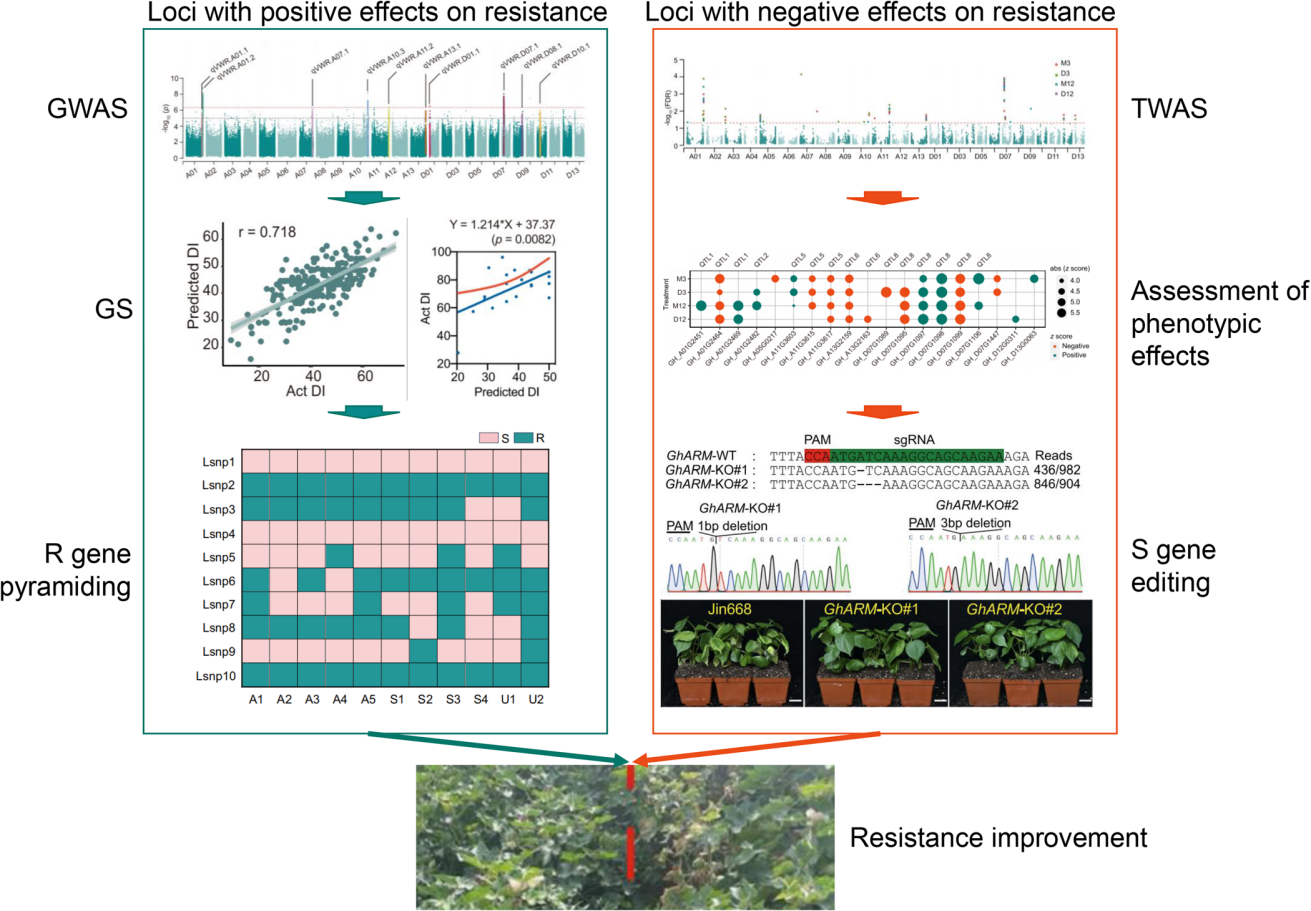
Illustration of identification and utilization of positive and negative effect loci associated with resistance traits. Left panel (positive effects): Genome‐wide association studies (GWAS) analysis identified quantitative trait loci (QTL) enhancing resistance and enabled phenotypic prediction based on genome selection (GS). Right panel (negative effects): Transcriptome‐wide association studies (TWAS) identified loci negatively impacting resistance, carried out the phenotypic effects assessment, and then carried out the S gene editing approaches for resistance improvement. The Figure is adopted from [3] DOI: 10.1002/imt2.70029.

S genes facilitate pathogen infection or suppress plant immunity. Editing S genes provides valuable targets for enhancing disease resistance [2]. For example, Wheat kinase *TaPsIPK1* is a susceptibility gene targeted by *Puccinia striiformis* effector PsSpg1. A CRISPR‐Cas9 edited allele of *TaPsIPK1* conferred robust rust resistance without growth or yield penalty both in the field and greenhouse [4]. However, loss‐of‐function mutations in S genes often incur fitness penalties [2]. Editing *MLO* in barley confers broad‐spectrum resistance to powdery mildew but induces growth retardation [5]. Similarly, *rod1* mutants enhance rice resistance to blast, sheath blight, and bacterial blight but reduce grain yield [6]. Introducing naturally adapted alleles or precise editing modifications may mitigate such trade‐offs [6, 7, 8].

## 1 ENHANCING CROP DISEASE RESISTANCE THROUGH PYRAMIDING OF RESISTANCE GENES

Compared with major gene‐mediated resistance, quantitative resistance controlled by minor gene is generally considered race‐nonspecific and more durable [9]. Pyramiding breeding employs molecular design to aggregate favorable alleles or functional quantitative trait loci (QTL) dispersed across diverse germplasm resources, constructing a synergistic genetic network. For instance, combining late blight resistance genes *Rpi‐vnt1.1*, *Rpi‐blb2*, *R8*, and *RB* in potato broadens the recognition spectrum against *Phytophthora infestans* and confers broad‐spectrum resistance [10]. In wheat, pyramiding resistance genes *Yr18*, *Yr28*, and *Yr36* in the highly susceptible line SY95‐71 confers sufficient stripe rust resistance, which translates to robust all‐stage resistance when introduced into elite lines at present [11]. Molecular marker‐assisted selection (MAS) has simplified our ability to combine Resistance genes/QTL. For example, pyramiding powdery mildew genes *Pm2*, *Pm4a*, and *Pm21* into the elite wheat cultivar “Yang158” through the molecular markers generated lines with broad‐spectrum resistance [12]. Similarly, pyramiding *Xa21* and *Xa27* based on MAS enhanced rice resistance to bacterial blight [13]. Advances in high‐throughput sequencing have enabled the simultaneous acquisition of extensive genetic markers, providing an ideal resource for MAS. Zhang et al. [3] identified 10 stable QTL by association analysis of Verticillium wilt resistance in natural cotton populations. Validation using lead SNPs (Lsnps) confirmed the pyramiding effects of these alleles (Lsnp^R^s) on the level of disease resistance across natural and artificial F_2:3_ populations, with resistance intensity correlating positively with the number of pyramided Lsnp^R^s [3]. This study offers a reference framework for allele pyramiding in the genomic era [3]. Modern pyramiding breeding marks a transition from “experience‐driven” to “design‐driven” crop improvement.

## 2 GENOME EDITING OF SUSCEPTIBILITY (S) GENES

In the study of Zhang et al., co‐suppression of eight negative regulators in cotton cultivar Zhongzhimian 2 (pyramided with 10 Lsnp^R^s) synergistically further enhanced Verticillium wilt resistance [3] (Figure 1). Integrating pyramided loci with positive effects (Lsnp1^R^, Lsnp4^R^, Lsnp5^R^, Lsnp8^R^, and Lsnp9^R^) alongside the targeted editing of loci with negative effects (*GhARM*) represents a viable strategy for cultivating durable and broad‐spectrum disease resistance in crops. Consequently, it addresses the limitations associated with single‐gene solutions. Given that pathogens are in a constant state of evolution, single‐gene strategies often lose their effectiveness over time. In contrast, this combined strategy offers a comprehensive and sustainable framework for safeguarding crops against pathogens that can rapidly adapt to their hosts.

Recently, the integration of the blast resistance gene *Piz‐t* with CRISPR/Cas9‐mediated knockout of S genes *Bsr‐d1*, *Pi21*, and *Xa5* generated the rice line 07GY31‐*BSR*, exhibiting BSR without growth penalties, proposes a novel strategy of targeted editing of S genes within a R gene background to develop broad‐spectrum disease‐resistant rice germplasm [14]. Furthermore, genomic selection (GS) models integrating multi‐omics (genome and transcriptome) provide a robust framework for accelerating the co‐improvement of disease resistance, yield, and quality. The study by Zhang et al. [3] established a comprehensive genetic and molecular framework for understanding Verticillium wilt resistance in cotton through a dual strategy—pyramiding elite alleles (denoted as “R” alleles, which demonstrated a statistically significant association with enhanced resistance to Verticillium wilt) and editing negative regulators. However, several limitations warrant acknowledgment to refine future research and breeding applications. First, this study primarily analyzed 290 Chinese upland cotton accessions, which may inadequately represent the global genetic diversity of cotton germplasm. Second, field trials were conducted in Xinjiang, China; their agroecological specificity and reliance on a single *V. dahliae* strain (V991) for seedling‐stage resistance evaluation limit the generalizability of findings across diverse environments and pathogen populations. Third, although pyramiding 10 QTLs reduced disease indices, the study did not systematically investigate potential trade‐offs with critical agronomic traits such as yield and fiber quality. Comprehensive field trials assessing pleiotropic effects are imperative to validate the breeding applicability of pyramided lines.

## 3 FUTURE DIRECTIONS AND CHALLENGES

Future breeding strategies challenges for crop disease resistance will include advanced genomic methods to: (1) address unintended negative effects of R gene pyramiding or S gene editing through precise optimization; (2) resolve interactions or redundancies in multi‐gene/QTL networks; (3) develop efficient delivery systems like Cas9‐PE [15] for multi‐gene editing; (4) rational deployment of resistance (R) and S gene combinations to mitigate pathogen evolution; (5) context‐specific selection of gene combinations across genetic backgrounds; (6) coordinate multi‐pathogen resistance gene introgression for broad‐spectrum varieties.

In summary, this study advances the genetic and molecular understanding of Verticillium wilt resistance in cotton and bridges basic research to breeding applications through multi‐omics integration and functional validation. As genomics and gene editing technologies converge, an era of “intelligent design” for disease‐resistant crops is rapidly approaching.

## AUTHOR CONTRIBUTIONS

**Qingdong Zeng**: Writing—review and editing; writing—original draft; project administration. **Zhensheng Kang**: Conceptualization; methodology; project administration; supervision.

## CONFLICT OF INTEREST STATEMENT

The authors declare no conflicts of interest.

## ETHICS STATEMENT

No animals or humans were involved in this study.

## Data Availability

Data sharing is not applicable to this article as no datasets were generated or analyzed during the current study. Supplementary materials (graphical abstract, slides, videos, Chinese translated version, and update materials) may be found in the online DOI or iMeta Science http://www.imeta.science/imetaomics/.

## References

[1] Savary, Serge , Laetitia Willocquet , Sarah Jane Pethybridge , Paul Esker , Neil McRoberts , and Andy Nelson . 2019. “The Global Burden of Pathogens and Pests on Major Food Crops.” Nature Ecology & Evolution 3: 430–439. 10.1038/s41559-018-0793-y 30718852

[2] Deng, Yiwen , Yuese Ning , Dong‐Lei Yang , Keran Zhai , Guo‐Liang Wang , and Zuhua He . 2020. “Molecular Basis of Disease Resistance and Perspectives on Breeding Strategies for Resistance Improvement in Crops.” Molecular Plant 13: 1402–1419. 10.1016/j.molp.2020.09.018 32979566

[3] Zhang, Xiaojun , Shiming Liu , Peng Wu , Wanying Xu , Dingyi Yang , Yuqing Ming , Shenghua Xiao , et al. 2025. “A Panoramic View of Cotton Resistance to Verticillium Dahliae: From Genetic Architectures to Precision Genomic Selection.” iMeta 4: e70029. 10.1002/imt2.70029 PMC12130556 40469521

[4] Wang, Ning , Chunlei Tang , Xin Fan , Mengying He , Pengfei Gan , Shan Zhang , Zeyu Hu , et al. 2022. “Inactivation of a Wheat Protein Kinase Gene Confers Broad‐Spectrum Resistance to Rust Fungi.” Cell 185: 2961–2974.e19. 10.1016/j.cell.2022.06.027 35839760

[5] Wang, Yanpeng , Xi Cheng , Qiwei Shan , Yi Zhang , Jinxing Liu , Caixia Gao , and Jin‐Long Qiu . 2014. “Simultaneous Editing of Three Homoeoalleles in Hexaploid Bread Wheat Confers Heritable Resistance to Powdery Mildew.” Nature Biotechnology 32: 947–951. 10.1038/nbt.2969 25038773

[6] Gao, Mingjun , Yang He , Xin Yin , Xiangbin Zhong , Bingxiao Yan , Yue Wu , Jin Chen , et al. 2021. “Ca^2+^ Sensor‐Mediated ROS Scavenging Suppresses Rice Immunity and Is Exploited by a Fungal Effector.” Cell 184: 5391–5404.e17. 10.1016/j.cell.2021.09.009 34597584

[7] Sha, Gan , Peng Sun , Xiaojing Kong , Xinyu Han , Qiping Sun , Laetitia Fouillen , Juan Zhao , et al. 2023. “Genome Editing of a Rice CDP‐DAG Synthase Confers Multipathogen Resistance.” Nature 618: 1017–1023. 10.1038/s41586-023-06205-2 PMC11575942 37316672

[8] Li, Shengnan , Dexing Lin , Yunwei Zhang , Min Deng , Yongxing Chen , Bin Lv , Boshu Li , et al. 2022. “Genome‐Edited Powdery Mildew Resistance in Wheat Without Growth Penalties.” Nature 602: 455–460. 10.1038/s41586-022-04395-9 35140403

[9] Li, Wei , Yiwen Deng , Yuese Ning , Zuhua He , and Guo‐Liang Wang . 2020. “Exploiting Broad‐Spectrum Disease Resistance in Crops: From Molecular Dissection to Breeding.” Annual Review of Plant Biology 71: 575–603. 10.1146/annurev-arplant-010720-022215 32197052

[10] Zhao, Xiaoqiang , Fan Zhang , Xiaoqing Chen , Chongyuan Zhang , Haoyi Zhang , Tian Wang , Jinzhe Zhang , et al. 2025. “Stacking Potato NLR Genes Activates a Calcium‐Dependent Protein Kinase and Confers Broad‐Spectrum Disease Resistance to Late Blight.” Journal of Integrative Plant Biology: 1–18. 10.1111/jipb.13892 40125812

[11] Wang, Fang , Minghu Zhang , Yanling Hu , Meijuan Gan , Bo Jiang , Ming Hao , Shunzong Ning , et al. 2023. “Pyramiding of Adult‐Plant Resistance Genes Enhances All‐Stage Resistance to Wheat Stripe Rust.” Plant Disease 107: 879–885. 10.1094/pdis-07-22-1716-re 36044366

[12] Liu, J. , D. Liu , W. Tao , W. Li , S. Wang , P. Chen , S. Cheng , and D. Gao . 2008. “Molecular Marker‐Facilitated Pyramiding of Different Genes for Powdery Mildew Resistance In Wheat.” Plant Breeding 119: 21–24. 10.1046/j.1439-0523.2000.00431.x

[13] Luo, Yanchang , Tingchen Ma , Aifang Zhang , Kar Hui Ong , Zhixiang Luo , Zefu Li , Jianbo Yang , and Zhongchao Yin . 2017. “Marker‐Assisted Breeding of Chinese Elite Rice Cultivar 9311 for Disease Resistance to Rice Blast and Bacterial Blight and Tolerance to Submergence.” Molecular Breeding 37: 106. 10.1007/s11032-017-0695-8

[14] Tao, Hui , Ning Xiao , Ruyi Wang , Feng He , Yue Cai , Su Jiang , Min Wang , et al. 2025. “Development of Elite Rice With Broad‐Spectrum Resistance Through Pyramiding of Key Resistance Gene and Simultaneously Editing Multiple Susceptibility Genes.” Journal of Integrative Plant Biology. 10.1111/jipb.13901 40136029

[15] Zou, Jinpeng , Xiangbing Meng , Zhengyuan Hong , Yuchun Rao , Kejian Wang , Jiayang Li , Hong Yu , and Chun Wang . 2025. “Cas9‐PE: A Robust Multiplex Gene Editing Tool for Simultaneous Precise Editing and Site‐Specific Random Mutation in Rice.” Trends in Biotechnology 43: 433–446. 10.1016/j.tibtech.2024.10.012 39537536

